# Age-appropriate vaccination against measles and DPT-3 in India – closing the gaps

**DOI:** 10.1186/1471-2458-13-358

**Published:** 2013-04-17

**Authors:** Niyi Awofeso, Anu Rammohan, Kazi Iqbal

**Affiliations:** 1 School of Population Health, University of Western Australia, Nedlands, Australia; 2 e-School of Health and Environmental Studies, Hamdan Bin Mohammed e-University, Dubai, United Arab Emirates; 3 School of Business, Discipline of Economics, University of Western Australia, Nedlands, Australia

**Keywords:** Measles, Vaccination schedule, India, Age-appropriate vaccination

## Abstract

**Background:**

In 2010, India accounted for 65,500 (47%) of the 139,300 measles-related deaths that occurred globally. Data on the quality of age-appropriate measles vaccination in rural India is sparse. We explored the following issues: (i) What proportion of Indian children were appropriately vaccinated against measles at 9 months of age, and DPT-3 at 4 months? (ii) Which health facilities administered measles vaccine to children prior to 9 months of age and DPT-3 prior to 14 weeks?

**Methods:**

We analyzed data from the 2008 Indian *District Level Health Survey* (DLHS-3) to determine the extent of age-appropriate measles and DPT-3 vaccinations. Among 192,969 households in the dataset, vaccination cards with detailed records were available for 18,670 children aged between 12 and 23 months.

**Results:**

Among this cohort, 72.4% (13,511 infants) had received the first dose of measles vaccine. Only 30% of vaccinated infants received the measles vaccine at the recommended age of 9 months. Similarly, only 31% of infants in the cohort received DPT-3 vaccine at the recommended age of 14 weeks. About 82% of all prematurely vaccinated children were vaccinated at health sub-centres, ICDS and Pulse Polio centres.

**Conclusions:**

Age-inappropriate vaccination impacts adversely on the effectiveness of India’s measles immunisation program due to sub-optimal seroconversion, if premature, and increased vulnerability to vaccine preventable diseases, if delayed. Capacity building approaches to improve age-appropriate vaccination are discussed.

## Background

It is generally advised that (except during an ongoing measles epidemic) children should not be vaccinated against measles until nine months of age, as vaccination prior to this may result in the neutralization of vaccine by maternal antibodies. In India and in most developing nations, the recommended time for administering the first dose of measles vaccine is 9 months [1]. In most developed nations, measles vaccine is typically administered at a minimum age of 12 months [2], although Australia’s government recently revised its childhood immunisation program to reschedule the first dose of measles-containing vaccine to 18 months (instead of 12 months) with effect from 1 July 2013. A major reason for deferring measles vaccine to at least 9 months of age is due to evidence of low measles post-vaccination seroconversion rates if vaccination is given before six months, and optimal seroconversion rates if vaccination is delayed until 12 months, with as low as 36% protection afforded by measles vaccination at 4–6 months of age [3]. Halsey *et al’s* landmark study of measles vaccine seroconversion among Haitian children, showed that seroconversion rate at 6 months of 45% increased to a seroconversion rate of 100% at 12 months [4]. While global coverage of DPT-3 has increased from 20% in 1980 to 83% in 2011, 32% of children who did not receive the DPT-3 as scheduled were from India [5]. Measles and DPT-3 vaccinations constitute reliable parameters for monitoring age-appropriate vaccination of childhood immunisation in India.

Although some researchers have suggested giving the first dose of measles vaccination at seven months [6] or giving vaccine doses at 4.5 months and then at 9 months in high prevalence regions, lowering the timing of first vaccination below 6 months has not been shown to significantly reduce all-cause mortality at age three years (at least among boys), compared with a single measles vaccine administered at 9 months following birth [7]. Moreover, logistic and cost implications of a two-part first measles dose strategy may be prohibitive for most poor nations who are currently unable to deliver the first dose of vaccine to over 90% of eligible children.

The level of immunity that children under nine months possess varies considerably depending on whether the mother acquired measles immunity via vaccination or natural infection [8-10], and on antibody variability in the infant [11]. Poor and undernourished children tend to acquire many infections at an early age, and passively acquired maternal antibody is neutralized earlier than among economically advantaged children, particularly in countries where low vaccine coverage does not offer herd immunity [11,12]. The 2008 *Indian District Level Health Survey* (DLHS-3) has documented that 67% of 1-year old rural Indian children have received first-dose measles immunization coverage, while the 2009 *Coverage Evaluation Survey* reported the national estimate for the first dose measles immunization coverage as being 74%, with 78% coverage documented for urban areas and 72% for rural areas [13]. The 29,808 estimated new cases of measles in 2010 and the 65,500 deaths from measles-related causes in India represent an unsatisfactory burden of disease reduction compared with the 2000 baseline [14]. However, both surveys relied largely on self-reports of measles vaccination to determine coverage rates. Studies on the reliability of mothers’ recall of measles vaccination have produced mixed results [15,16]. As the mothers’ recall data from the DHLS-3 does not include information on the timing of measles vaccination, we were limited in this study to data available from vaccination cards. Health workers’ documentations however, provide objective evidence of vaccination and reduce the possibility of recall errors.

Interestingly, although the 2009 Indian measles immunization coverage of 74% is close to the average measles coverage for the South East Asia region of 78%, India accounted for 58% of all measles cases in the South-East Asian region in 2009 [17]. The fact that measles outbreaks continue to occur in Indian districts with high measles vaccination coverage [18] highlight the adverse impact of low vaccine efficacy on measles control. Apart from reduction or loss of vaccine potency due to the failure of the cold chain system [19], low vaccination coverage (particularly among vulnerable populations such as slum dwellers) and inadequate surveillance, age-inappropriate vaccination is a major constraint in optimising measles and DPT vaccination outcomes in India [20,21]. The Millennium Development Goal indicator 4.3 for measles immunization coverage refers to the proportion of one-year old children (i.e. 12 – 23 months old) immunized against measles [22]. In 2008, the Indian National Technical Advisory Group on Immunization recommended the introduction of a second dose of measles-containing vaccine, delivered through routine vaccination in states with ≥80% coverage with the first dose of measles-containing vaccine, or through mass vaccination campaigns in states with <80% measles containing vaccine coverage. Based on these recommendations, the Government of India initiated a second-dose measles vaccination in late 2010, to be given anytime from eight weeks following the first dose [1]. However, progress is slow not only because only a minority of Indian districts have achieved over 80% measles vaccination coverage, but also because global measles funding has declined from $US150 million in 2007 to $US35 million in 2010, leading to postponement of scheduled vaccination campaigns [23].

Given the critical influence of the timing of vaccination on measles incidence and mortality, we analysed the timing of measles vaccination among a sample of Indian children with vaccination cards, using the nationally representative DLHS-3 dataset. To examine if findings on age-appropriateness of measles vaccination apply to other vaccinations, we analysed the age-appropriateness of the Diphtheria-Pertussis-Tetanus – 3^rd^ dose (DPT-3) vaccination. This vaccine is scheduled to be administered at 14 weeks, according to India’s vaccination schedule [1]. We sought to answer the following questions: (i) What proportion of Indian children aged over 12 months were administered the first dose of measles at 9 months of age, and the third dose of DPT at 4 months of age? (ii) For children that were administered the first dose of measles vaccine prior to 9 months of age, and the third dose of DPT prior to 4 months of age, which health facilities administered the vaccine? (iii) How can monitoring of measles vaccination be improved to ensure that vaccination is administered age-appropriately?

## Methods

The 2008 DLHS-3 was collected by the International Institute for Population Sciences, Mumbai on behalf of the Ministry of Health and Family Welfare, Government of India. All data were de-identified. The de-identified version of the DLHS-3 data is publicly available upon formal request for access to the Director of India’s Institute of Population Sciences. As no patient could be identified or contacted, no ethics approval was required by individual researchers to undertake this study.

The Immunization and Child Care Section (Section 3) of the DLHS-3 survey records immunization history of the last two surviving children. It also contains information on the place of vaccination. All women who had given birth in the five years prior to the survey were asked about the immunization status of their last two surviving children. Using this data, we determined the age of the child at the time of vaccination, and the place of the vaccination.

There are 46,362 children aged between 12–23 months in DLHS-3, of whom 18,670 (40%) were able to show their vaccination card (recorded by Indian health workers), 14,451 (31%) claimed to have a card but were unable to produce them and the remaining 13,281 (29%) did not have a card. As the dataset did not include mothers’ self-reports of timing of their children’s vaccinations, only data on the 18,670 children with filled out cards were used to determine age-appropriateness, and the place where measles and DPT-3 vaccinations were administered.

## Results

Of the 18,670 children with verifiable vaccination cards, 72% received the first dose of measles vaccination. This figure is identical to that obtained in the 2009 Coverage Evaluation Survey, but higher than the 68% vaccination coverage when self-reported vaccinations are included. The vaccination coverage is also much lower than the 83% global measles vaccination coverage in 2008 [14]. Similar trends were noted for DPT-3 vaccination. Of 18,670 eligible children, 82% were vaccinated with DPT-3, compared with 72% DPT-3 vaccination rate in the 2009 Coverage Evaluation Survey [13], and 83% global DPT-3 vaccination coverage in 2011 [5].

Table 1 shows the proportion of children in our sample vaccinated against measles at specified months. Only 30% of one-year olds received the first dose of measles vaccine at the appropriate age of 9 months.

**Table 1:** Timing of measles vaccination – DLHS-3

**Vaccination age**	**No. of children**	**Percent**	**Cumulative percent**
0	71	0.53	0.53
1	41	0.3	0.83
2	56	0.41	1.24
3	65	0.48	1.72
4	87	0.64	2.37
5	117	0.87	3.23
6	158	1.17	4.4
7	327	2.42	6.82
8	1,084	8.02	14.85
9	4,011	29.69	44.53
10	3,608	26.7	71.24
11	1,599	11.83	83.07
12	872	6.45	89.53
13	473	3.5	93.03
14	286	2.12	95.14
15	219	1.62	96.77
16	123	0.91	97.68
17	97	0.72	98.39
18	75	0.56	98.95
19	54	0.4	99.35
20	42	0.31	99.66
21	29	0.21	99.87
22	14	0.1	99.98
23	3	0.02	100
Total	13,511	100	

Source: DLHS-3.

Apart from the 28% of the children in the sample who were not vaccinated against measles, about 7% of children in the sample received the first dose of measles vaccine after 12 months of age. During this period, such children are vulnerable to measles infection as they lack maternal antibodies. Furthermore, a high proportion of rural-based Indian infants are malnourished, thus making them more vulnerable to measles infection. Since most Indian regions have generally low measles vaccination coverage (< 85%), the median age of measles infection is low, and the median attack rate of measles is high among these children [24]. Similar age-inappropriate trends were observed for DPT-3 vaccination (Table 2), with only 31% vaccinated with DPT-3 in the fourth month as recommended by India’s childhood immunization schedule. About 14% of all children with documented cards were given DPT at or prior to, the 12 week, although the vaccine was due to be administered by the 14^th^ week, and preferably no later than the 16^th^ week [1].

**Table 2:** DPT-3 vaccination by age, in months

**Vaccination age**	**No. of children**	**Percent**	**Cumulative percent**
0	58	0.38	0.38
1	78	0.51	0.89
2	336	2.19	3.07
3	1,671	10.88	13.95
4	4,767	31.03	44.98
5	3,187	20.75	65.73
6	1,753	11.41	77.14
7	982	6.39	83.54
8	658	4.28	87.82
9	499	3.25	91.07
10	356	2.32	93.39
11	255	1.66	95.05
12	209	1.36	96.41
13	126	0.82	97.23
14	103	0.67	97.9
15	94	0.61	98.51
16	92	0.6	99.11
17	42	0.27	99.38
18	45	0.29	99.67
19	18	0.12	99.79
20	22	0.14	99.93
21	8	0.05	99.99
23	2	0.01	100
Total	15,361	100	

Source: DLHS-3.

Indian health workers involved with vaccination programs are expected to be familiar with the vaccination schedule, and not vaccinate any child against measles prior to nine months of age or, for DPT-3, prior to the 4^th^ month.

Data on the place of measles vaccination presented in Table 3 show that 82% of all prematurely administered vaccinations were delivered at three venues: the Sub-centers, Integrated Child Development Services (i.e. *Anganwadi*) and Pulse Polio Centers. Of the 4,011 children that received the age-appropriate vaccinations at 9 months of age, only 55% were vaccinated at these centres.

**Table 3:** Percentage of children vaccinated prematurely against measles by place of vaccination

**Place of vaccination**	**Percentage vaccinated (13511)**	**Early vaccination Age < 9**
*Public health facilities*		
ICDS centre (Anganwadi)	36.61	38.58
Sub-centre	27.04	24.75
PHC	17.40	17.57
Hospital	10.80	8.80
CHC/Rural hospital	5.50	4.67
Dispensary	2.30	1.62
Other public health facility	0.93	1.04
Mobile clinic	0.77	1.10
UHC/UHP/UFWC	0.60	0.84
NGO/Trust Hospital/Clinic	0.32	0.31
Ayush hospital/clinic	0.22	0.20
*Private Health facilities*		
Pulse Polio	13.86	18.19
Other private health facilities	6.83	2.80
Private hospital	5.55	4.56
Private doctor/clinic	1.19	1.36
Pharmacy/drug store	0.19	0.20
Private Ayush hospital/clinic	0.04	0

Source: DLHS-3.

Table 3 shows that competency deficiencies (assessed in this study by proportion of prematurely vaccinated children) affect both the public and private health sectors in India, but are most marked amongst the typically less technically qualified staff (including volunteers) who work in Sub-health centres, Pulse Polio and *Anganwadi* centers [25,26]. Age-inappropriate vaccination for measles is a major health risk for affected children. Parents of children vaccinated before six months of age are lulled into a false sense of security, although their children remain at risk of measles infection. Failure to vaccinate against measles not only increases the risk of measles-related morbidity and mortality, but may also significantly increase the risk of all-cause mortality [27]. The unsatisfactory level of measles vaccination documentation – less than 20% of one year olds in DLHS-3 have filled out vaccination cards - is a crucial indicator of poor measles vaccination surveillance in India [28,29].

## Discussion

Our review reveals three important weaknesses in India’s childhood vaccination program: (i) poor quality surveillance and documentation of childhood immunization data; (ii) sub-standard indicators for vaccination quality monitoring (the extent that MDG indicator for adequacy of measles immunisation currently in use is not sensitive enough to determine premature measles vaccination as well as delayed vaccination prior to 23 months); (iii) inadequate training, competency, or supervision of some of the staff administering measles vaccination, as evidenced by the majority of premature vaccination taking place in centres where health workforce is least trained. For example, a Sub-health centre is typically staffed by one Female Health Worker commonly known as Auxiliary Nurse Midwife (ANM) and one Male Health Worker commonly known as Multi-Purpose Worker. Although there are documented minimum standards of care expected of staff at the Sub-health centres [30], there is no minimum standard of training or qualifications required to work in sub-health centres, the most peripheral and first contact point between the primary health care system and the community.

Optimising age-appropriate vaccination is a major priority for effective delivery of immunizations in India [31,32]. Data on India’s measles statistics from the South-East Asian Regional office, reveals that there have been no formal surveillance studies on nationwide measles morbidity and mortality since 2005 [30]. Poor documentation of vaccination data inevitably results in erroneous estimates. While the WHO data focusses on children immunized by 12– 23 months of age, vaccination given prior to 6 months of age is very likely to be of sub-optimal efficacy - studies indicate that 64% of measles vaccine recipients at 4–6 months of age are vulnerable to infection due to neutralization by maternal antibodies [3]. Children immunized following 12 months of birth are not protected by maternal antibodies after one year of birth, and are vulnerable to wild measles infection, given the absence of herd immunity [33]. The longer such immunization is delayed, the greater is the risk of severe measles infection.

In rural India, with 72% of the vulnerable population immunized against measles, it is not surprising that case fatality rate is high, and the average age of infection is concentrated in the first three years of life [34]. Similarly, given that child morbidity (India reported 26,044 cases of pertussis in 2005) and mortality from pertussis is high among infected children who have not completed their third dose of vaccination prior to six months of age [35,36], the fact that only 77% of children received the third dose of DPT vaccine by the end of the sixth month (Table 2) highlights the potential adverse health consequences of delays in timing childhood immunizations in India.

The MDG indicator 4.3 for measles immunization coverage [22] fails to distinguish between those vaccinated prior to six months, those vaccinated between 12 and 23 months, and those vaccinated in age-appropriately. It is acknowledged that the MDG indicators were consensus instruments designed to optimise ease of measurement and global acceptance, however, as measles prevalence falls and the 2015 post MDG era approaches, it is important to refine this indicator in order to improve program outcomes. A more sensitive age-appropriate indicator of measles immunization coverage might read; “the percentage of children aged 12 – 23 months who were vaccinated at 9 months of age”.

Our data indicates that coverage with first dose of measles vaccine was ≥ 90% in only 26% of the evaluated Indian districts. State-level coverage for the first dose of measles vaccine ranges between 48% to 96% [33]. The wide variability in program outcomes mirrors asymmetries in the quality of measles vaccination program management in various districts of India.

Table 3 shows that 38.6% of all premature measles vaccinations (i.e. vaccination prior to the age of 9 months) took place in Integrated Child Development Centers (i.e. *Anganwadi*), and maybe in part due to their inadequate education and competency level, and in line with their sub-optimal performance in the nutrition and health education components of the program [37]. Apart from very low seroconversion rates, premature administration of measles vaccine may also result in infants receiving DPT vaccination following measles vaccination, a factor associated with high female mortality [38]. This may imply that the task shifting of measles vaccination activities to the least trained and supervised health staff (who vaccinated 77% of all children against measles in the analysed data) may be compromising the quality of measles vaccination in India, thus contributing to the current unsatisfactory trends in measles vaccination and consequently high measles morbidity and mortality.

India’s Universal Immunisation program has been performing sub-optimally over the past decade, with average national immunisation coverage in 2009 reported as being 61%, and a wide range of vaccination coverage between states, the highest coverage recorded in Goa (88%) and the lowest in Nagaland (27%) [39]. The reasons for poor age-specific timing of measles vaccination are also related to India’s poorly functioning public health system [40,41].

This study has several limitations. First, it is focused on children with documented vaccination cards as this is the only cohort for whom there is information on the timing of vaccination. We acknowledge that the exclusion of children with no documented vaccination cards may limit the applicability of our findings to the general Indian population. Second, we have focused on service factors affecting sub-optimal vaccination. While it is possible that structural determinants such as gender, socio-economic factors and access to vaccination centres may influence age-inappropriate vaccination, nevertheless, we believe that the impact of such social determinants would deviate from the key point that we want to make in this paper on the crucial role of the service providers in vaccinating appropriately. Third, we have no data on the training, supervision or competencies of staff in Sub-health centres, Pulse Polio centres and ICDS centres during the period DLHS-3 data were collected in those centres. Our observations and comments on these centres are based on secondary data [42] as well as on field visits in 2012 to a sample of these centres, and may therefore differ from the actual workforce and program management situation in these centres in 2008 when data for our study were collected.

## Conclusion

This study reveals that India’s 2008 measles vaccination coverage in rural areas was 72%, based on documented information from vaccination cards. Among those vaccinated, age-inappropriate vaccination is common and a major factor contributing to India’s disproportionately high measles morbidity and mortality in 2010. Age-inappropriate vaccination practices associated with measles are reflected in DPT-3 vaccination, making it likely that such deficiencies affect all childhood vaccinations. We suggest a three-pronged approach to improve age appropriateness of measles immunization in India; improving surveillance and documentation systems so as to better organize childhood immunization for children at the optimal age range; refining measles vaccination coverage indicators to better define the proportion of children who have received the first dose of measles vaccine at the appropriate of 9 months, and; improving measles vaccination program management and supervision, particularly in the locations where vaccines are being delivered prematurely, as well as in the 74% of districts with less than 90% immunization coverage by age 23 months. The successful eradication of polio in India in 2012 highlights the possibilities for measles eradication if concerted efforts are made to improve the quality and coverage of measles immunization. Assuring age appropriate measles vaccination is an important quality indicator in this regard.

## Competing interests

The authors declare that they have no competing interests.

## Authors’ contributions

AR and NA conceptualized the study. AR, NA and KI conducted data analysis. NA conducted literature review and worked with AR and KI to interpret data. All authors read and approved the final manuscript.

## Pre-publication history

The pre-publication history for this paper can be accessed here:

http://www.biomedcentral.com/1471-2458/13/358/prepub
